# ^18^F-FDG PET/CT detection of primary mammary-like adenocarcinoma of the vulva with multiple metastases: a case report

**DOI:** 10.3389/fonc.2024.1441064

**Published:** 2024-11-14

**Authors:** Qing Song, Biyun Zhang, Yu Gu

**Affiliations:** ^1^ The First School of Clinical Medicine, Nanjing University of Chinese Medicine, Nanjing, China; ^2^ Department of Nuclear Medicine, Affiliated Hospital of Nanjing University of Chinese Medicine, Nanjing, China; ^3^ Department of Oncology, Affiliated Hospital of Nanjing University of Chinese Medicine, Nanjing, China

**Keywords:** mammary-like adenocarcinoma, vulvar adenocarcinoma, FDG, PET/CT, vulvar cancer

## Abstract

Mammary-like adenocarcinoma of the vulva is a malignancy with a low incidence rate compared with the squamous cell carcinoma occurring at the same site. We present a rare case of mammary-like adenocarcinoma of the vulva with multiple-organ involvement using ^18^F-FDG PET/CT. This study indicates that ^18^F-FDG PET/CT can not only detect the primary lesion but also distinguish the stage of the mammary-like adenocarcinoma of the vulva.

## Introduction

The mammary-like adenocarcinoma (MLA) of the vulva is a rare primary vulvar tumor occurring in ectopic vulvar breast tissue (1), and it shows a low incidence rate, and has immunohistochemical profiles and aggressive behaviors similar to those of breast cancers, which make it challenging for clinical diagnosis and differential diagnosis. PET/CT plays an important role in the management of vulvar cancer (2–4). However, at present, there is little literature on the detection of MLA of the vulva with ^18^F-FDG PET/CT imaging.

In this case, the ^18^F-FDG PET/CT imaging indicated a malignant tumor according to morphological manifestations and metabolic characteristics, which was definitely diagnosed as primary MLA of the vulva by the pathological examination.

## Case description

**Figure 1:**
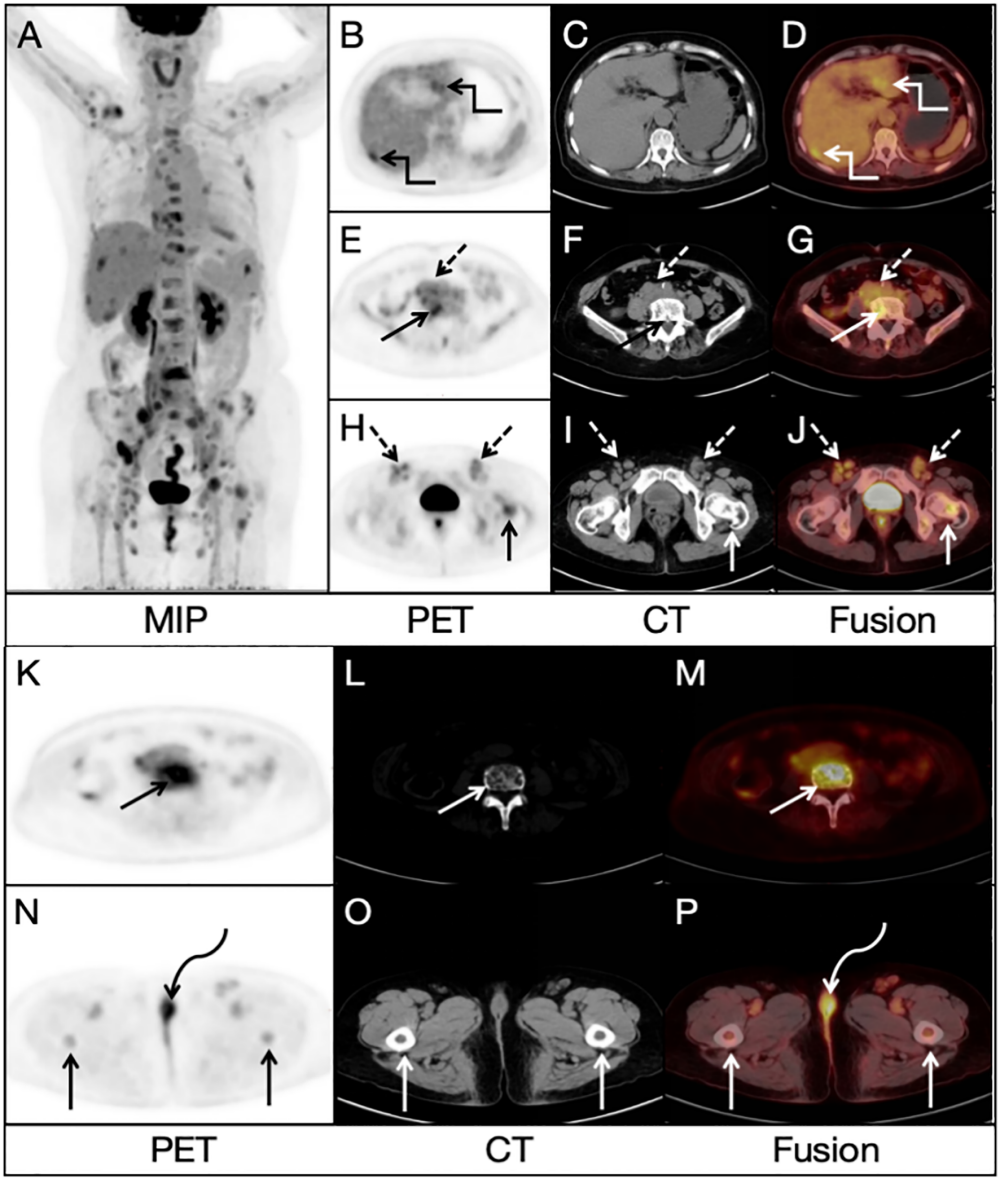
Radiological findings on ^18^FDG PET/CT image: **(A)** Multiple lesions in the bones, liver and lymph nodes. **(B, D)** Multiple foci of abnormal activity (broken arrows) in the liver. **(C)** No clear evidence of anatomical abnormality in the corresponding area on CT image. **(E–J)** Enlarged retroperitoneal and bilateral inguinal lymph nodes (dashed arrows). **(K–M)** Multiple hypermetabolic foci (solid arrows) in the bones **(N, P)** An additional focal activity (curved arrows) with a SUV_max_ of 6.2 in the vulva. **(O)** No clear evidence of anatomical abnormality in the corresponding area on CT image.

A 68-year-old woman presented with low back pain for 1 month, and a non-contrast-enhanced abdominal CT revealed several lesions on her spine bones, suggesting multiple metastatic lesions. Furthermore, laboratory examinations showed increased tumor markers: CA125: 50.7 U/ml (normal value < 35 U/ml), Ferritin: 539 ng/ml (normal value 13-150 ng/ml). This patient had no previous history of malignancy. For evaluating the general condition, this patient underwent ^18^F-FDG PET/CT imaging, which demonstrated multiple fluorodeoxyglucose (FDG) uptake in the liver, bones and lymph nodes in the PET/CT MIP image (**Figure 1A**). Multiple foci of abnormal activity (broken arrows) were seen in the liver (**Figures 1B, D**), although there was no clear evidence of anatomical abnormality in the corresponding CT image (**Figure 1C**). Multiple hypermetabolic foci (solid arrows) were also seen in the bones (**Figures 1E–P**), with mixed bone destruction on CT (**Figure 1L**). Enlarged retroperitoneal and bilateral inguinal lymph nodes (dashed arrows) with increased activities were seen on PET/CT (**Figures 1E–J**). An additional focal activity (**Figures 1N, P**, curved arrows) with SUV_max_ of 6.2 was also observed in the vulva, although CT indicated no clear evidence of anatomical abnormality in the corresponding area (**Figure 1O**).

**Figure 2:**
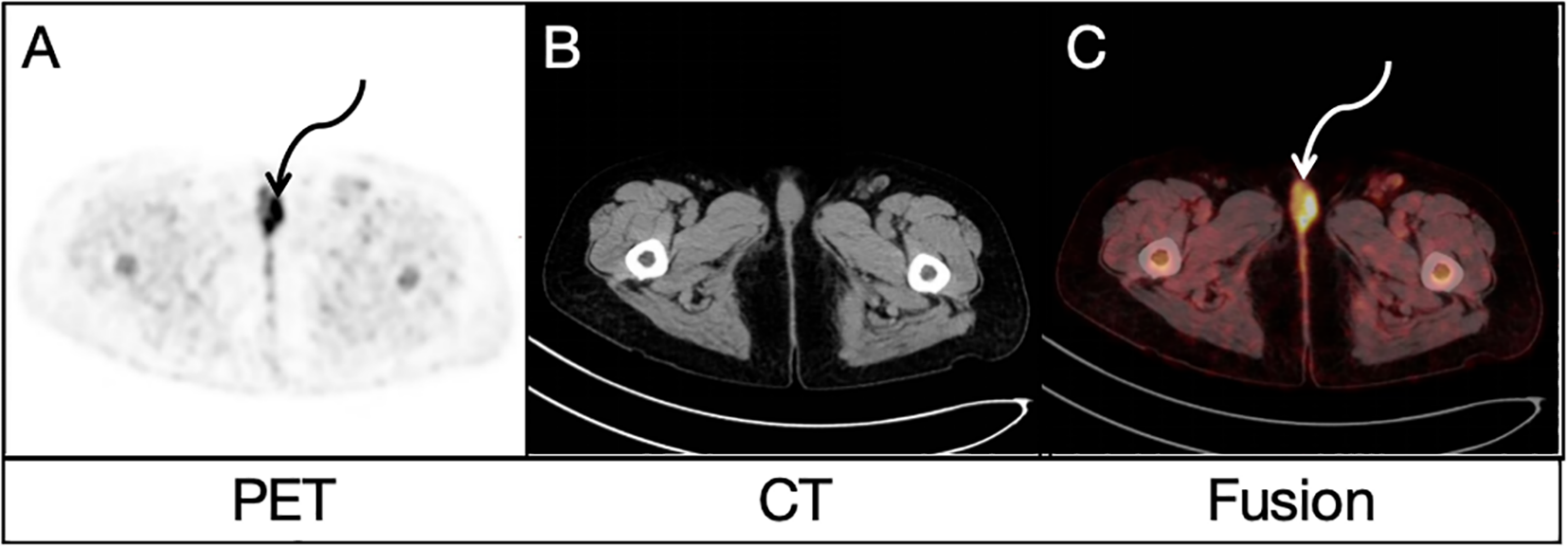
Delayed pelvic FDG PET/CT findings: Both PET image **(A)** and PET/CT fusion image **(C)** demonstrated similar possible malignant lesions (curved arrows) in the vulva, with a SUVmax of 10.2 while no evidence of anatomical abnormality in the corresponding area on CT image **(B)**.

FDG uptake in many lesions in the liver, bones and lymph nodes might suggest multiple metastases, but the location of primary lesion was unclear. Due to the existence of bilateral inguinal lymph node metastases, the vulvar lesion was suspected to be the primary lesion. To further clarify the diagnosis, this patient underwent a delayed pelvic FDG PET/CT imaging at 2 h after FDG administration, and the results demonstrated multiple possible malignant lesions in the vulva (**Figures 2A, C**, curved arrows), with a furtherly increased SUV^max^ of 10.2 while no evidence of anatomical abnormality in the corresponding area on CT image (**Figure 2B**). A more detailed medical history was then taken. The woman had been complaining of vulvar inflammation and pain for years. The physical examination had revealed the presence of stiffness and swelling in the mons pubis, accompanied by two erythematous lesions of 1.0 cm in diameter. According to the medical history and findings of dual-time-point ^18^F-FDG PET/CT, vulvar carcinoma with liver, bone and lymph node metastasis was suggested.

**Figure 3:**
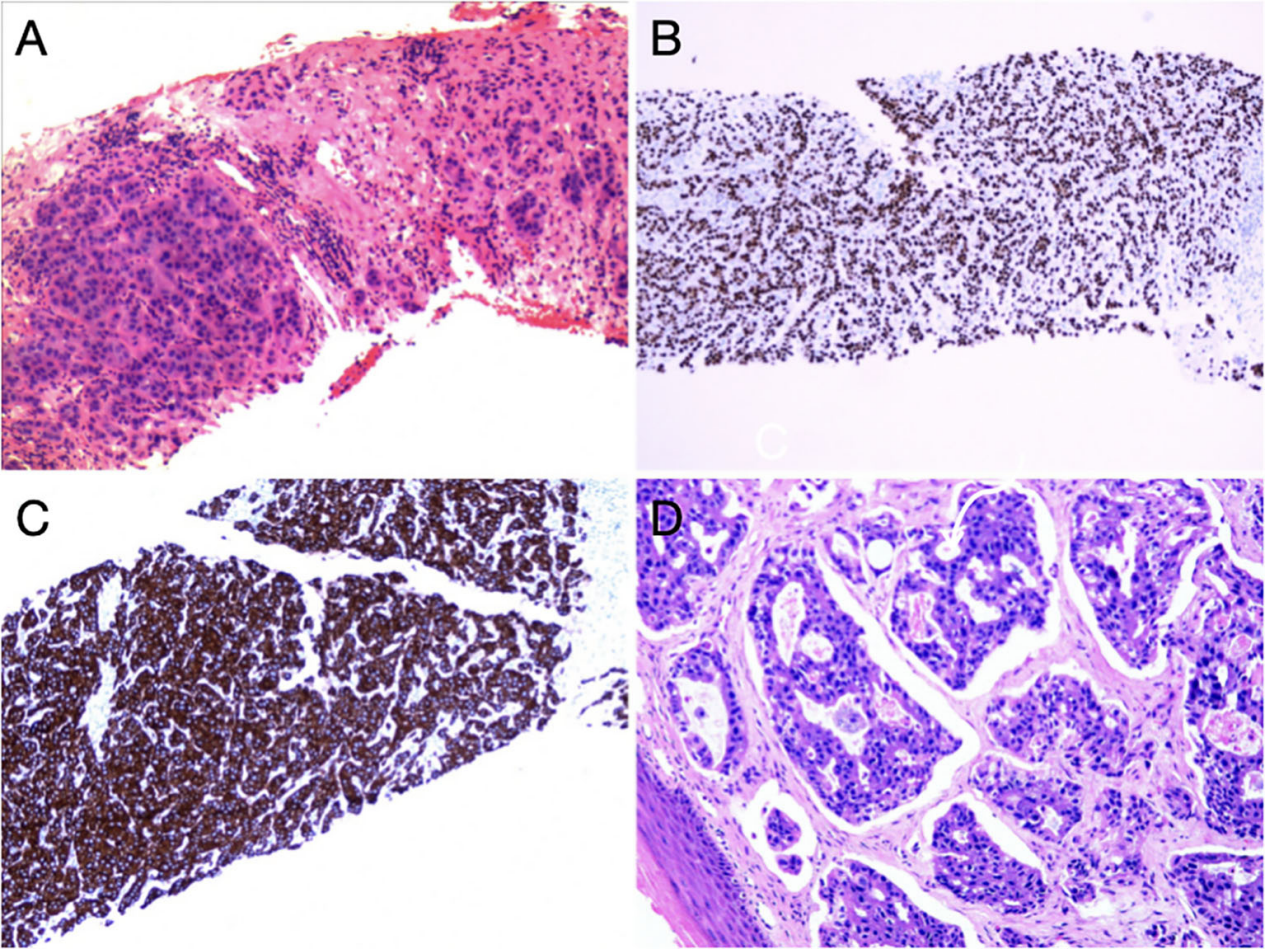
Pathohistological findings of inguinal lymph nodes and vulva: **(A)** HE staining of right inguinal lymph nodes. In fibrous connective tissue and lymphoid tissue, there were nests and cords of heterogeneous cells with enlarged nuclei and an increased nucleo-plasmatic ratio, and these features were consistent with those of malignant epithelial tumors. **(B)** Positive expression of GATA3. **(C)** Strongly positive expression of CK7. **(D)** HE staining of the vulva. There were cribriform hyperplasia and nest-shaped clusters of cells, local necrosis and vascular cancer thrombus in the tissues.

Subsequently, biopsy and pathological examination of affected vulva (**Figure 3D**) and right inguinal lymph nodes (**Figures 3A–C**) were performed, indicating that the vulvar lesion was an infiltrative, moderately/poorly differentiated adenocarcinoma arising from mammary gland-like epithelium of the vulva. In addition, the immunohistochemical results showed that the estrogen receptor/progesterone receptor and positive common breast cancer markers such as epithelial membrane antigen (EMA), carcinoembryonic antigen (CEA), cytokeratin 7(CK7), GATA-binding protein 3(GATA3) and Ki-67 were increasingly expressed by more than 40%. The morphology and immunoprofile of the tumor cells suggested the incidence of MLA.

This patient had no history of breast malignancy, and no abnormal ^18^F-FDG uptake was observed in bilateral breasts. Finally, this patient was diagnosed with a primary MLA of the vulva, with multiple metastases in liver, bones and lymph nodes. At present, the patient is receiving palliative radiotherapy in local lesion of vulvar MLA (with a prescription dose of 50 Gy in 25 fractions) after 6 cycles of chemotherapy with paclitaxel and carboplatin. The outcome after chemotherapy was assessed as stable.

## Discussion

It has been reported that the squamous cell carcinoma is the most common malignant tumor in the vulva, and accounts for 76% of vulvar cancers, while the adenocarcinoma only accounts for less than 2% of vulvar cancers (5). Van der Linden et al. (6) have observed that the majority of glandular malignancies in the vulva are primary tumors, which account for 55%. Primary adenocarcinomas in the vulva predominantly include extramammary Paget’s disease and sweat gland carcinomas. In addition, a rare form of adenocarcinoma is known as MLA of the vulva, which originates from putative mammary-like glands of the vulva, and exhibits a spectrum of pathological reactions that are similar to those in their mammary counterparts (7–9). Notably, the extramammary Paget’s disease can also present as a manifestation of underlying breast carcinoma, thus contributing to the differential diagnosis (10). The diagnosis of vulvar malignancies is obviously dependent on pathological evaluation (11). Since vulvar MLA and breast cancer have morphological similarities and similar aggressive behaviors, the criteria for diagnosing vulvar malignancy were defined by referring to breast carcinoma (12): in the absence of concurrent breast carcinoma, a primary adenocarcinoma of mammary-like glands of the vulva can be diagnosed when its morphological pattern is consistent with that of the breast carcinoma, with the presence of estrogen/progesterone receptors and positive markers such as CK 7 and GATA 3, which are common in breast cancers. In this case, the immunohistochemical results were measured against the above-mentioned criteria.

It is worth noting that HER-2, as one of the molecular typing markers of breast cancer, theoretically has an important reference value in the diagnosis and treatment of vulvar MLA (13). Unfortunately, the HER-2 expression was not detected in this case. Among previous reports (14), HER-2 expression was detected in a few cases, indicating a potential value of HER-2 in the diagnosis and treatment of vulvar MLA.

Ki67 is widely recognized as the best indicator of cell proliferation activity. Rolfe et al. (15) demonstrated that the increased expression of Ki67 reflects the changes in the proliferation during vulvar carcinogenesis, suggesting that Ki67 can serve as an indicator of tumor invasiveness. This patient is still under treatment, and attention will be paid to the correlation between tumor progression and ki67 expression. However, a study (16) has shown that Ki67 has no prognostic value in patients with vulvar Paget’s disease (PDV) or mammary Paget’s disease (PDB). Therefore, further study is needed to explore the correlation between Ki67 expression and this disease in the future.

The MLA originating from the vulva is exceedingly uncommon, and fewer than 40 cases have been reported since 1935 (14, 17). Only two patients in these reports underwent FDG PET/CT to assess the stage of the adenocarcinoma, and the results revealed that one patient had no metastatic disease, while the other patient exhibited distant metastases in lymph nodes and bones (18, 19). PET/CT plays an important role in the management of vulvar cancer. In the case reported by Patel D et al. (20), PET/CT exerted an important effect in staging of an identified vulvar cancer. Moreover, Triumbari EKA et al. (21) demonstrated that a negative preoperative PET/CT imaging may indicate no groin metastases in early-stage vulvar cancer patients, thus it’s not necessary for them to undergo sentinel node biopsy. And Treglia G et al. (22) reported a case of vulvar extramammary Paget’s disease (EMPD) restaged by PET/CT. In this case, PET/CT scan was helpful in locating the primary tumor. The manifestations of some inflammatory lesions and tumors on PET/CT are similar. Zhuang H et al. (23) have proved that the SUVs of delayed images of malignant lesions increase over time compared with those of earlier images, while the SUVs of the inflammatory lesions and benign lesions remain stable or decrease slightly over time. Another clinical trial (24) also showed that dual-time-point FDG-PET imaging has a potential to improve the accuracy of distinguishing between inflammation and tumor lesions. In this case, the manifestations of vulvar lesions on dual-time-point FDG PET/CT clearly showed this feature, and the SUV_max_ increased from 6.2 on the initial image to 10.2 on the delayed image. Furthermore, some studies (25, 26) have shown that the radiomics features of PET/CT images have an important value in lymph node assessment and prognosis prediction in patients with vulvar cancers.

Because of the rarity of this disease and the lack of definitive treatment guidelines, this type of cancers is currently staged and treated according to the treatment method for primary breast cancers (27, 28). Surgical excision with adjuvant therapies such as radiation, anthracycline-based chemotherapy and hormonal therapy is a common option (29). Benito V et al. (30) presented a case of an elderly patient with metastatic vulvar adenocarcinoma arising from mammary-like glands successfully treated with a combination of surgery and hormonal therapy. And Tanaka H et al. (31) reported a case of MLA successfully treated by paclitaxel weekly without excision. In addition, Butler B et al. (32) explored the application of sentinel lymph node mapping in patients with MLA, this technique has been almost exclusively applied in patients with breast carcinoma. It is very suitable to use sentinel lymph node mapping in the patients with vulvar cancers, which can predict the lymphatic drainage of the vulva, with a lower false negative rate and a substantially reduced adverse reaction rate (e.g. lymphedema) compared with the total groin lymph node dissection (33, 34). Tessier-Cloutier B et al. (35) observed that the breast carcinoma and MLA of the vulva had similar intrinsic luminal molecular subtypes, and thus proposed a new treatment strategy, that is, molecular subtyping of breast cancer can be performed to optimize individual treatment.

To sum up, we report a rare case of MLA of the vulva with multiple-organ involvement detected by ^18^F-FDG PET/CT. In this case, according to the distribution characteristics of lesions on PET/CT, the primary tumor was found, indicating the important role of PET/CT. Furthermore, dual-time-point PET/CT imaging demonstrates a great diagnostic value and potential significance in differential diagnosis. The effects of PET/CT imaging in prognostic prediction of MLA remain to be confirmed in future studies. This case is described based on the real world, and there are some deficiencies in the diagnosis of MLA of the vulva, such as insufficient collection of detailed medical histories and a lack of physical examination before PET/CT imaging, which have increased the difficulty of diagnosis. The study of this case reveals that optimized diagnostic strategy and appropriate imaging technique are especially important in the clinical diagnosis of rare diseases.

## Data Availability

The original contributions presented in the study are included in the article/supplementary material. Further inquiries can be directed to the corresponding author.
